# Investigation of two different PACAP-38 (Pituitary Adenylate Cyclase Activating Polypeptide) formulated feeds on Atlantic salmon (*Salmo salar*) immune responses with Enteric Red Mouth disease (*Yersinia ruckeri*)

**DOI:** 10.1016/j.cirep.2025.200221

**Published:** 2025-03-30

**Authors:** E Fajei, L Rivera Méndez, SK Whyte, J Velazquez, P Dantagnan, M Soto-Davila, T Rodríguez-Ramos, E Proskos, B Dixon, Y Carpio, M Estrada, MD Fast

**Affiliations:** a Department of Pathology and Microbiology, Atlantic Veterinary College, University of Prince Edward Island, Charlottetown, PEI, Canada; b University of Waterloo, Waterloo, ON, Canada; c Veterinary Immunology Project, Animal Biotechnology Division, Center for Genetic Engineering and Biotechnology, Havana, Cuba; d Department of Agricultural Sciences and Aquaculture, Faculty of Natural Resources, Catholic University of Temuco, Temuco, Chile

**Keywords:** Salmon, PACAP-38, Enteric Red Mouth disease, Antimicrobial resistance (AMR), Antimicrobial peptide (AMP)

## Abstract

In salmonids, as in other teleosts, Pituitary Adenylate Cyclase Activating Polypeptide (PACAP) has been demonstrated to have anti-inflammatory activity, anti-oxidative stress, and cytoprotective impacts against several aquatic pathogens, including *Yersinia ruckeri*, which is the causative agent of Enteric Red Mouth (ERM) disease. This study aimed to evaluate the impacts of feed formulated with two different forms of PACAP-38 on the immunological response of Atlantic salmon (*Salmo salar*) to infection with *Y. ruckeri*. Atlantic salmon (*n* = 318, 45±5.6 g) were randomly assigned to three replicate tanks per diet, with a commercial base diet (Placebo), an amidated form of PACAP added to the commercial diet, and a non-amidated form of PACAP added to the commercial diet. After 28 days of feeding these diets, two tanks per diet underwent bath exposure (80 L tank for 60 min with 72 fish; water temperature was 16.6 ± 0.2 °C) to *Y. ruckeri* (isolate U27451–11; at 7.8 × 10^8^ CFU/ml) and one tank per diet was unexposed. Fish (five fish per tank) were sampled prior to exposure, 1 day, 3 days, 7 days, and 20 days post-infection (dpi). Infected salmon that received amidated PACAP feed showed a significantly higher survival rate (*P=0.006*) than the Placebo group. Bacteriological samples collected from the kidney and intestine identified *Y. ruckeri* as the cause of death. Acute mortality coincided with strong inflammatory responses that were variable across fish and groups. Furthermore, PACAP diets induced some inflammatory gene expression in the head kidney, intestine and spleen, mostly at 3 and 7 dpi (*il10 and hepc*). These data suggest that both amidated and non-amidated PACAP had induced *il1ß, hepc and il10*, and the timing suggests protection against infection and stress, possibly by inhibiting inflammatory pathways and reducing oxidative stress. Due to the anti-inflammatory effects of *il10* and its pleiotropic nature, gene expression results in response to PACAP feeds may suggest a more complex regulation of inflammation in the PACAP-fed fish. Further, *mmp9* gene expression and assessment of IgT and IgM in the serum were observed to be stimulated earlier and maintained longer in the amidated diet, contributing to the resolution of infection and reduction of mortality.

## Highlights

• ELSA detection of antigen-specific IgT and IgM levels revealed that infected fish receiving both non-amidated and amidated PACAP had significantly lower IgT levels in their serum compared to those on a placebo diet.
• Additionally, a significant difference was noted between the amidated and non-amidated groups within the non-infected category after quantifying IL-1β serum protein concentrations at 1 day post-infection (dpi).
• Notably, in the gut, there were marked differences in *il-1β* expression among the three diets at both 1 dpi and 3 dpi. Furthermore, in infected fish, *il-10* expression showed significant downregulation in the gut tissue at 1 dpi in the non-amidated PACAP group.
• When comparing the expression of *hepcidin* between infected and non-infected groups in the spleen and head kidney at 1 dpi, significant upregulation was observed in the infected groups for both amidated and non-amidated PACAP in the spleen. Moreover, the spleen demonstrated a significant increase in *tgf-β* expression following infection with *Yersinia ruckeri* in the amidated PACAP group.
• The amidated PACAP-38 feed elicited greater immunological responses in all three tissues at all time points and resulted in decreased mortality rates.

## Introduction

According to the status quo, aquaculture is the food industry with the fastest rate of growth, with 185.5 million tons of production worldwide in 2023 [1]. This food sector can support several million people's livelihoods globally and contributes considerably to nutrition and food sustainability, especially in some of the most food-insecure regions. Asia accounted for 80 % of the world's aquaculture production in terms of quantity and economic value in 2018, with aquaculture contributing 46 % of the world's total fish production. To secure the growing human population in 2050, the Food and Agriculture Organization of the United Nations (FAO) predicted that there would need to be a 70 % increase in the global supply of food and feed [2]. Bacterial infections and disease outbreaks are significant issues for intensive fish farming due to severe economic loss owing to morbidity and mortality [3]. The emergence of antimicrobial resistance (AMR) bacteria in aquaculture has become a serious concern, with 90 % of bacteria in the aquatic environment exhibiting resistance against at least one antibiotic [4]. Recently, Geetha et al. 2020 ranked Enterobacteriaceae third in terms of importance among pathogens causing mortality in aquaculture that demonstrate AMR [5]. Therefore, looking for suitable alternative therapeutic methods is crucial.

There are numerous bacterial diseases in fish, such as Aeromonas septicemia, Columnaris, Vibriosis and Enteric Red Mouth (ERM) disease, which have been reported in the aquaculture sector [6]. ERM is a bacterial infection that can be isolated from marine and freshwater species on farms and wildlife. It severely impacts salmon farming by drastic mortality, with chemotherapeutic intervention being the most effective treatment for this disease [7]. *Y. ruckeri*, a freshwater pathogen, is the causative agent of this disease and results in several external and internal signs in fish, including exophthalmos, darkening and subcutaneous hemorrhage in the mouth and throat, which is indicative of the disease and the term enteric red mouth [8].

Antibiotic therapy has been widely used for decades to cure these illnesses in aquaculture worldwide [9]. According to previous records, approximately 73 % of the top 15 aquaculture-producing countries utilize oxytetracycline, florfenicol, and sulphadiazine as the primary treatment [10]. Moreover, the growing volume of this usage over the past ten years has broadened the evidence of (AMR) [11] and made finfish aquaculture, with its rapid expansion, more susceptible to microorganisms [12]. Therefore, the usage of other immunoprophylactic measures for controlling disease, including vaccinology, pro-and prebiotic treatments, and immunostimulants that can prime both innate and adaptive immune systems, has expanded in the industry.

Antimicrobial peptides (AMPs) and proteins are a diverse novel class of molecules considered as a substitution for antibiotics [13,14]. Many AMPs have been found in humans, animals, plants, bacteria and fungi. These peptides play a pivotal role in the body's primary response against invasion, highlighting their importance in the immune system, particularly innate immunity [15].

Pituitary Adenylate Cyclase Activating Polypeptide (PACAP) is an evolutionarily conserved AMP produced in the brain in response to bacterial and fungal infections and extensively distributed throughout organs, including the digestive system [16]. Expression of the PACAP-specific receptor, PAC1-R, in Grass carp *(Ctenopharyngodon idella)* head kidney leucocytes, provides evidence that PACAP may directly affect fish immune cells [17]. PACAP can perform a variety of regulatory roles and cytoprotective effects in the body through the process of binding with many different G protein-coupled receptors, including PAC1 (PAC1-R), VPAC1 (VPAC1-R), and VPAC2 (VPAC2-R) receptors on innate immune cells, for instance, macrophages and lymphocytes [18]. It has also demonstrated its role in reducing oxidative stress, cellular infiltration, apoptosis, and its anti-inflammatory effects [19]. Oral administration of therapeutic substances is one of the ways drugs are delivered to fish. It is less invasive and stressful, less labor-intensive, and suitable for mass administration to fish of all sizes. Furthermore, vaccines and drugs administered in the feed are totally non-stressful ways that do not interfere with routine husbandry practices [20]. However, previous studies have shown that intraperitoneal (i.p.) injection of PACAP-38 elicits a stronger immune response compared to other administration routes [21].

This research examines the impacts of two forms of feed formulated with PACAP-38 on subsequent infection with *Y. ruckeri* in Atlantic salmon (*Salmo salar*) immunophysiological responses.

## Methods

### Fish maintenance and husbandry

Atlantic salmon were purchased from Kelly Cove Salmon Ltd, New Brunswick, Canada and shipped to the Aquatic Biological Containment Level II Facility at the Atlantic Veterinary College (AVC), Charlottetown, PE. After 14 days of acclimation, 318 (45±5.6 g) fish were randomly assigned to nine tanks (approximately 35 fish/ tank) (270 L) (three replicate tanks per diet) and maintained in a single pass, freshwater flow-through aquatic system at 12 ± 1 °C. Dissolved oxygen (DO) was maintained above 80 % saturation and pH 7.5–7.8. Throughout the study (two months), fish were hand-fed at 2 % per body weight over two feedings per day, and uneaten pellets obtained after flushing ∼20 L water from the tanks were counted and recorded. Fish were maintained under a 12h:12 h light-dark photoperiod. Salmon were kept off feed 24 h before handling. The UPEI animal care committee approved all protocols and procedures, following the Canadian Council of Animal Care guidelines (AUP protocol #19–050). At the end of the study, all remaining fish were euthanized by tricaine methane sulfonate (TMS) overdose (300 mg/L).

### Culture of *Y. ruckeri*

*Y. ruckeri* isolate U27541–11 (originated from an Atlantic salmon submitted to the Aquatic diagnostic lab at AVC-UPEI, presenting external signs of ERM; 22) culture was started on a tryptone soy agar (TSA) plate from frozen stock and incubated at room temperature (22 °C) for 24 h to check for purity and viability. After one day, a colony was transferred to 20 mL of tryptone soy broth (TSB) and incubated for 15 h on the shaker at 22 °C. The following day, ten flasks were inoculated with 5 mL of overnight culture, each containing 600 mL of TSB, resulting in a starting OD600 of 0.048. Those flasks were incubated on the shaker (125 rpm) at room temperature for 6 h to reach an OD600 of 1.8 (at the beginning of the log phase). The bacterial culture was prepared to have 7.8 × 10^8^ CFU/ml as the final concentration in the tanks.

### Feed production and formulation process

*Clarias gariepinus* synthetic PACAP-38 (amino acid sequence, HSDGIFTDSYSRYRKQMAVKKYLAAVLGRRYRQRFRNK) non-amidated variant was purchased from CS Bio (Shanghai) Ltd, China (non-amidated PACAP). The amidated form of PACAP-38 was synthesized at the Center for Genetic Engineering and Biotechnology, Havana, Cuba (amidated-PACAP). According to previous work [23], three different pre-formulations varying only in the type of synthetic PACAP-38 or Placebo (all the components without PACAP) were prepared as water-in-oil (W/O) emulsions. Three extruded diets were formulated and manufactured with Placebo or PACAP (0 or 250 μg of PACAP-38 per kg of feed) at the Animal Feed Pilot Plant at the Department of Agricultural and Aquaculture Sciences, Catholic University of Temuco, Chile. PACAP W/O pre-formulations were included in each experimental diet during the oiling phase of feed pellets using a vacuum coater (Dinnissen model VC10, Sevenum, Netherlands), according to the previous research [23].

### Study design

This research was designed to examine the immunological responses of Atlantic salmon exposed to *Y. ruckeri*. A total of 106 fish (45±5.6 g) were randomly distributed to each of three replicate tanks per diet. Experimental diets included the two types of PACAP-modified feed (amidated and non-amidated-PACAP) and a commercial base feed with a Placebo formulation that acted as a control group. After 28 days of feeding, fish were exposed to *Y. ruckeri* (7.8 × 10^8^ CFU/ml)in a bath challenge (80 L tank for 60 min; water temperature was 16.6 ± 0.2 °C; Dissolved Oxygen (DO)85–100 %). One tank per diet (three tanks) was not challenged with *Y. ruckeri* and served as a non-challenged negative control.

### Sampling

Fish were sampled five times over a period of 20 days. The first sampling occurred after 28 days of experimental feeding and prior to bacterial exposure. Subsequent samplings occured for 1 day, 3 days, 7 days, and 20 days post-infection (dpi). At each sampling point, three to five fish per tank were euthanized with an overdose of TMS (300 mg/L) and sampled. The tissues collected for gene expression analysis were a portion of the hindgut, head kidney, spleen and a minimum of 1 mL of blood. The serum was extracted from blood samples following 1-hour refrigeration. For histological purposes, small parts of the distal intestine and head kidney were sampled and placed in 10 % neutral buffered formalin for at least 24 h before embedding for sectioning and staining with Hematoxylin and Eosin (*H* + *E*). Microbiological samples (*n* = 24; *n* = 16 from the kidney and *n* = 8 from the intestine) were collected from moribund fish and cultured on TSA media to confirm the cause of death. Identification of the recovered bacteria was conducted by Diagnostic Services, UPEI, using a MALDI-TOF MS [22].

### Quantitative assessment of gene expression

RNA was extracted from the head kidney (*n* = 168), spleen and intestine (*n* = 138 per samples) samples using Tri-Reagent, following the Chomczynski & Sacchi [24] protocol. Ambion TURBO DNA-free™ treatment was used as instructed by the manufacturer to remove the remaining genomic DNA impurity. RNA integrity and quantity were examined utilizing BioRad's Experion™ Automated Electrophoresis Station (run on 1.5 % agarose in 1X TAE for 35 min in 100v on Mupid) and ThermoFisher's Nanodrop 3000. All the samples had RNA Quality Indicator (RQI) values >6, and 260/280ratio between 1.8 and 2.0).

The RNA molecules were reverse transcribed using BioRad's iScript™ Reverse Transcription cDNA kit. The reaction specifications were as follows: 2 µg of RNA, 8 µl of 5x iScript™ RT supermix, and nuclease-free water were used to make a total volume of 40 µl. The Eppendorf MasterCycler thermal cycler synthesized cDNAs with the following steps: priming at 25 °C for 5 min, reverse transcription (RT) at 46 °C for 20 min, and RT inactivation at 95 °C for 1 min. Quantitative Polymerase Chain Reaction (qPCR) was performed on BioRad's CFX384 thermal cycler, utilizing the following thermal regime: 95 °C for 30 s (1 cycle), 95 °C for 15 s, then 60 °C for 30 s (40 cycles), followed by melt curve analyses from 65–95 °C with fluorescence being read every 0.5 s with a ramp rate of 0.5 °C. To test primer efficiency, a pooled sample of each tissue was made by combining 2 µl aliquots of each RNA sample before cDNA synthesis. Standard curves were generated for each gene tested to assess primer efficiency, using the pooled sample to perform serial dilutions. Two reference genes and different genes of interest [Table 1; [18], [19], [20]] (*gyrB*, of *Y. ruckeri* kindly gifted by Dr. Eslamloo Table 1) were tested on the head kidney, spleen and gut cDNA samples. Their quality was also checked with melting point analysis (i.e., one peak in the melt curve). Each reaction (12 µl total volume) consisted of 5 µl of SsoAdvanced Universal ™ SYBR ® Green Supermix (BioRad), 2 µl cDNA template, 4 µl of nuclease-free water, and 0.5 µl of both forward and reverse primers10 µM. Each sample was run in triplicate, and no RT (no reverse transcriptase control) was also included for each gene. To control sample contamination, every plate had non-template control (NTC) and positive control (a pool of all samples) to calibrate across plates. Any Cq values greater than 38 cycles were considered non-specific amplification due to the cycles' proximity to the NTC. Triplicate Cq values per sample were used to determine the level of immunological responses, where lower Cq values were indicative of a higher level of responses to the treatment. Replicate group Cq standard deviations were not greater than 0.5.

**Table 1:** Primer sequence and PCR efficiency table for Atlantic salmon *(Salmo salar)*.

Genes of Interest	Forward Primer (5′−3′)	Reverse Primer (3′−5′)	Efficiency for Head-kidney	Efficiency for Spleen	Efficiency for Gut
Interleukin-1 beta (*il1ß*)^A^	ATGCGTCACATTGCCAAC	GGTCCTTGTCCTTGAACTCG	93.5	N/A	N/A
Interleukin-1 beta (*il1ß*)	CCACAAAGTGCATTTGAAC	GCAACCTCCTCTAGGTGC	N/A	104.2	105.6
Interleukin-6 beta (*il6ß*)	CCTTGCGGAACCAACAGTTTC	CCTCAGCAACCTTCATCTGGTC	N/A	107.8	N/A
Interleukin-10 (*il10*)	GGGTGTCACGCTATGGACAG	TGTTTCCGATGGAGTCGATG	94.4	110.7	103.5
Hepcidin *(hepC)*	GATGTGTACCCTGTGGCTAA	GTAGATTCCATTCCCAGACC	82.5	94.05	N/A
Transforming Growth Factor-beta *(tgfß)*	AGTTGCCTTGTGATTGTGGGA	CTCTTCAGTAGTGGTTTGTCG	N/A	91.9	N/A
Matrix Metalloproteinase-9 *(mmp9)*^B^	AGTCTACGGTAGCAGCAATGAAGG	CGTCAAAGGTCTGGTAGGAGCGTAT	100.7	N/A	N/A
Complement C3a	ACTTCGCCATACACCATCCT	GTCACATCAATCTCCACTCCAC	94.8	N/A	N/A
Tapasin *(tapbp)*	GGAGGCCACTTCTCCTCTCT	GTCTAGCCCCGCTCTCTTTT	91.8	N/A	N/A
Tumor Necrosis Factor-alpha (*tnfα)*	GGCGAGCATACCACTCCTCT	TCGGACTCAGCATCACCGTA	N/A	99.2	108.5
Interferon-gamma (*ifny*)	GAAGGCTCTGTCCGAGTTCA	TGTGTGATTTGAGCCTCTGG	N/A	110	106.2
Reference genes	**Forward Primer (5′−3′)**	**Reverse Primer (3′−5′)**	**Efficiency for Head-kidney**	**Efficiency for Spleen**	**Efficiency for Gut**
Ribosomal protein S20 (*rps20*)^C^	GCAGACCTTATCCGTGGAGCTA	TGGTGATGCGCAGAGTCTTG	95.4	N/A	N/A
Elongation Factor 1 Alpha (*ef1a-b*)^C^	TGCCCCTCCAGGATGTCTAC	CACGGCCCACAGGTACTG	94	N/A	N/A
Elongation Factor 1 Alpha (*ef1a-b*)	CGCACAGTAACACCGAAACGAATTAAGC	GCCTCCGCACTTGTAGATCAGATG	N/A	97.2	95.7
Beta-actin (*ß-actin*)	TGGACTTTGAGCAGGAGATGG	AGGAAGGAGGGCTGGAAGAG	N/A	94.9	99.2
Yersinia primer	**Forward Primer (5′−3′)**	**Reverse Primer (3′−5′)**	**Efficiency** **for Head-kidney**	**Efficiency for Spleen**	**Efficiency for Gut**
YR-DNA gyrase subunit B *(gyrB)*	CCCAACCGGTATGCATGATG	AGCCGCCAGAAACTTTGTAC	108 %	N/A	N/A

Where necessary (low expressing genes), PCR spike-ins were prepared to establish a standard curve. PCR products for each assay were made using 12.5 µl GoTaq colourless, 0.5 µl of forward primers, 0.5 µl of reverse primer, 2 µl from cDNA template and 8.5 µl of nuclease-free water and run on an Eppendorf MasterCycler thermal cycle. The program was: initial heating at 95 °C for 2 min, then denaturation at 95 °C for 1 min, annealing at 60 °C for 30 s, extension at 72 °C for 30 s, repeat these last three steps for 40 cycles, final extension at 72 °C for 5 min, and hold at 4 °C. PCR products were then run on 2 % agarose gel in 1X TAE at 100 V for 35 min to confirm if the samples were positive or negative for all genes. Gels were viewed on a UV Transilluminator at 302 nm.

### Histology examination and scoring

The histological scoring system was designed such that the lowest score was associated with no lesion observed in the tissues; score 1 showed focal mild injuries, score 2 indicated multifocal moderate, and score 3 presented diffuse severe damage to the tissues [25]. The expected tissue injuries were goblet cell hyperplasia, lymphangiectasia, edema and shortening of villi in the intestines or inflammation, necrosis and bacteria and cell infiltration in the kidney.

### IL1ß and IFN-γ quantification by sandwich ELISA

The concentration of IL-1β and INF-γ in the fish serum was determined following the protocol of Frenette et al. (2023), Soto-Dávila et al. (2024) and Rivera Méndez et al. (2024) [[26], [27], [28]].

### Determination of antigen-specific IgT and IgM levels by indirect ELISA

IgT and IgM anti-*Y. ruckeri* in the serum samples were measured by indirect ELISA following Soto-Dávila et al. (2024) protocol [27]. Samples were run in triplicate and consisted of 100 µL of serum diluted 1/3 in PBS (Corning, USA).

### Statistical analysis

The qPCR results were imported to qbase + software to normalize and calibrate relative quantities based on particular reference genes. Using one-way and two-way ANOVA following Tukey HSD analyses, relative gene expression values were analyzed using Graph Pad (Prism 10), which has also been used for visualization and performing Kaplan-Meier survivor analysis. Scores from the histological analysis were visualized in bar charts with multiple panels using Minitab Statistical Software Version 21.1.0. Also, the Kruskal-Wallis Test has been utilized to analyze the histological scores of tissue damage among treatment groups of infected fish.

## Results

### Survival rate and clinical signs of infection

**Fig. 1:**
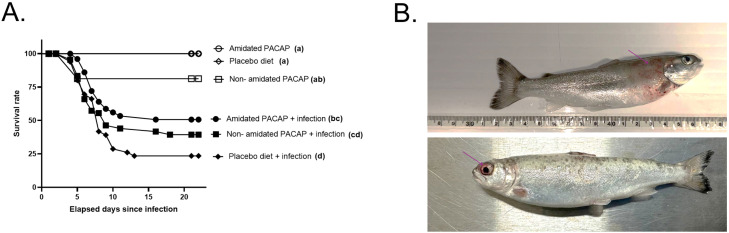
Survival curves of each group fed three different diets, followed by *Y. ruckeri* exposure (A). In infected fish, subcutaneous regions with petechial hemorrhage were identified with a purple arrow (B).

This study recorded mortality 20 days after infection, and a Kaplan-Meier survivor analysis was performed. The infected non-amidated showed no significant differences in survival with the amidated (*P* *=* *0.13*) or the infected control (*P* *=* *0.1*) groups. However, the infected amidated group demonstrated a significantly higher survival rate than the infected placebo diet (*P* *=* *0.006*). It is worth noting that the uninfected groups did not show significantly different mortality rates, and the unexpected mortality was observed in the non-amidated PACAP group (Fig. 1A).

The fish exposed to *Y. ruckeri* after receiving the experimental feed for 28 days showed clinical signs of ERM (Fig. 1B), such as exophthalmos, subcutaneous hemorrhagic congestion, and petechial hemorrhage on the fin and visceral surface [29]. Furthermore, the morphological features of the colonies on all the TSA plates had characteristics consistent with colonies of *Y. ruckeri:* yellow, opaque colonies with approximately 2–3 mm in diameter were smooth and slightly convex [30]. All colonies tested on the MALDI-TOF were confirmed to be *Y. ruckeri.* The culture and MALDI confirmation were reliable, however non-quantitative. Therefore, *Y. ruckeri* primers (*gyrB*, Table 1;) were tested on all the head kidney samples (*n* = 122). The pool of all cDNA samples amplified with conventional PCR consistently achieved a high concentration (Ct= 17–18), and while all uninfected samples were negative, only four infected fish tissue samples from all treatment groups were quantifiable (Ct values between 31–36). Therefore, quantification of *Y. ruckeri* infection was not pursued further.

**Fig. 2:**
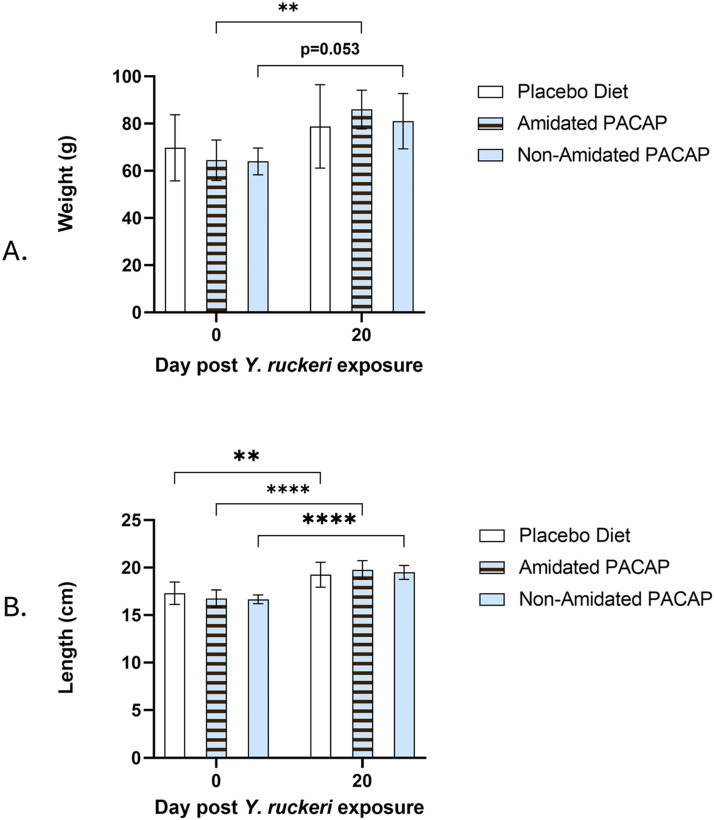
Comparing the weight (g) (A) and length (cm) (B) of fish in all three diets after 20 days post-infection and asterisks denoted the significant difference among groups (*P < 0.05, **P < 0.01, ***P < 0.001, ****P < 0.0001).

Moreover, the weights and lengths of the infected fish were compared at 0 dpi and 20 dpi between all diets. The analysis showed a significant increase in the weight of the fish in the amidated groups (*p* *=* *0.007*, Fig. 2A). Comparison of the length of the infected fish after 20 days post-infection demonstrated a significant acceleration in amidated, non-amidated and placebo diets (*p* *<* *0.0001, p* *<* *0.0001, p* *=* *0.0037* respectively; Fig. 2B) but higher on PACAP-fed groups.

### Gene expression analysis

**Fig. 3:**
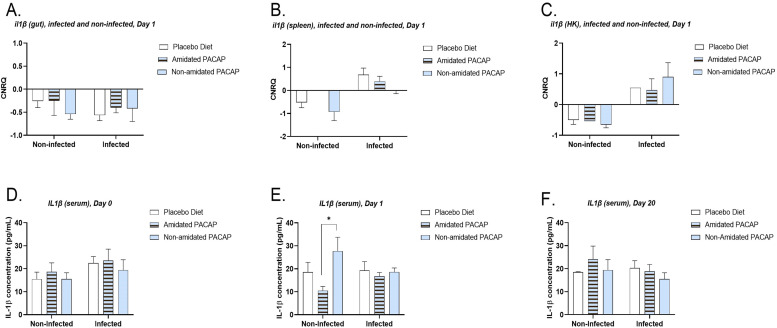
Calibrated normalized relative quantity of gut (A), spleen (B) and head kidney (C) *il1ß* expressions of Atlantic salmon (*Salmo salar*) at Day 1 are shown with bars. Bars show the mean ± SEM. The protein concentration of IL1 ß (pg/mL) in serum was measured and compared among three diets on Day 0 (D), Day 1 (E), and Day 20 (F).

**Fig. 4:**
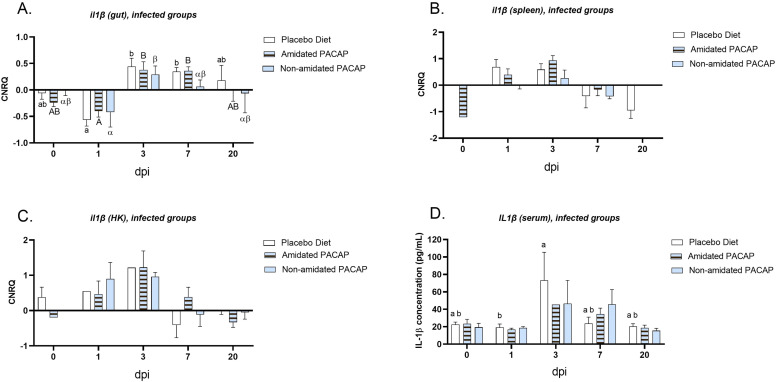
Calibrated normalized relative quantity of gut (A), spleen (B) and head kidney (C) *il1ß* expressions of infected Atlantic salmon (*Salmo salar*) at all time points are shown with bars. Letters denote significant group differences (*p<0.05*). The protein concentration of IL1 ß (pg/mL) in the serum of infected fish was measured and compared among three diets at all time points (D). Bars show the mean ± SEM.

**Fig. 5:**
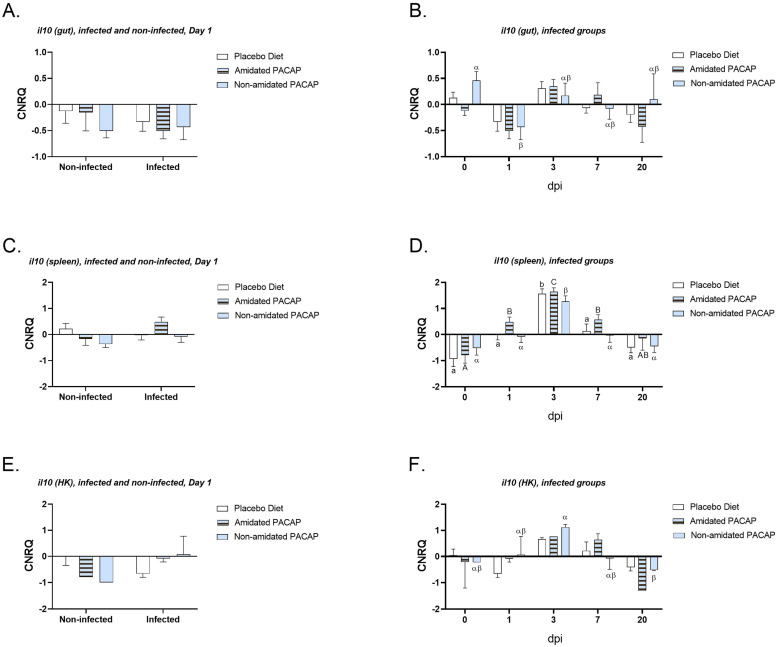
Calibrated normalized relative quantity of gut (A), spleen (C) and head kidney (E) *il10* expressions of Atlantic salmon (*Salmo salar*) at Day 1 are shown with bars. Calibrated normalized relative quantity of gut (B), spleen (D) and head kidney (E) *il10* expressions of infected Atlantic salmon (*Salmo salar*) at all time points are shown with bars. Bars show the mean ± SEM. Letters denote significant group differences (*p<0.05*).

**Fig. 6:**
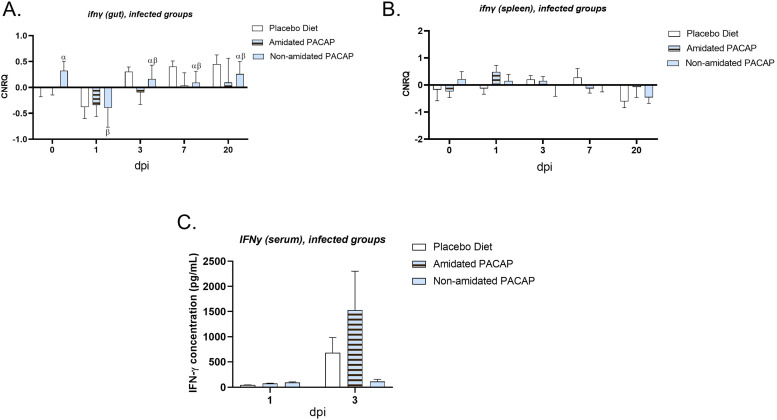
Calibrated normalized relative quantity of gut (A), spleen (B) *inf*γ expressions of infected Atlantic salmon (*Salmo salar*) at all time points are shown with bars. Bars show the mean ± SEM. Letters denote significant group differences (*p<0.05*). The protein concentration of INFγ (pg/mL) in the serum of infected fish was measured and compared among three diets at all time points (C).

To investigate the immunological responses to infection and the potential effects of PACAP-supplemented feed on gene expression, ten genes of interest (GOIs) were evaluated across three tissue types. It is important to note that it was not possible to test all GOIs in each tissue. The expression of *il1ß* was analyzed across all diets in infected and non-infected fish at 1-day post-infection (dpi) for the spleen, gut, and head kidney tissues (Fig. 3A, B and C). However, no significant differences were observed among the groups. A more detailed analysis of *il1ß* expression in the gut, spleen, and head kidney among infected groups was conducted at all time points (Fig. 4A, B, and C, respectively). In the gut, significant differences in *il1ß* expression were found between the three diets on Day 1 and Day 3, as well as between Day 1 and Day 7. However, no significant difference was observed between the expression of *il1ß* on Day 3 and Day 7. Additionally, the expression data for *il10*, which was tested in gut, spleen, and head kidney samples, indicated no significant difference between infected and non-infected groups at 1 dpi (Fig. 5A, C, and E). Nevertheless, in infected fish, *il10* expression showed significant downregulation in the gut (Fig. 5B) with non-amidated PACAP treatment in the first 24 h (*p* *=* *0.03*). In the spleen (Fig. 5D), *il10* expression in infected groups significantly (*p* *<* *0.0001*) increased during the first three days post-infection for all three diets. However, the expression of *il10* in the head kidney (Fig. 5F) in infected fish of non-amidated PACAP significantly *(p* *=* *0.009)* decreased between 1 and 20 dpi. The expression of *ifnγ* was measured in the intestine and spleen of infected fish over 20 days post-infection (Fig. 6A and B), revealing significant downregulation, although analysis indicated no significant difference in its expression in the gut at Day 1.

**Fig. 7:**
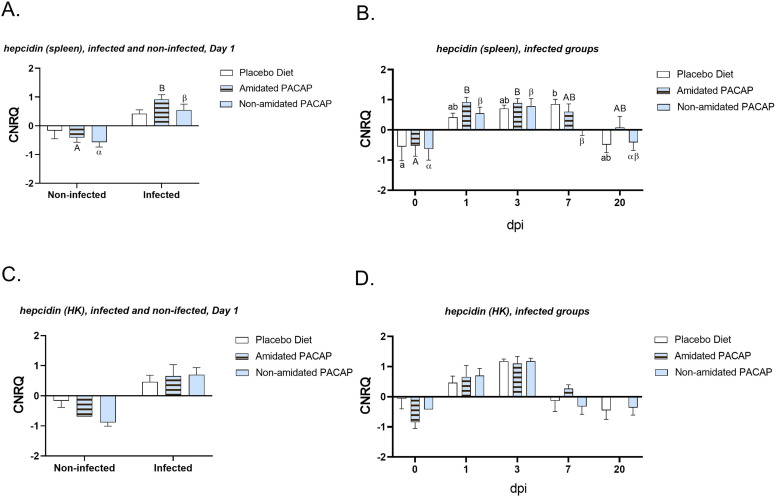
Calibrated normalized relative quantity of spleen (A) and head kidney (C) *hepcidin* expressions of Atlantic salmon (*Salmo salar*) at Day 1 are shown with bars. Calibrated normalized relative quantity of spleen (B), head kidney (D) *hepcidine* expressions of infected Atlantic salmon (*Salmo salar*) at all time points are shown with bars. Bars show the mean ± SEM. Letters denote significant group differences (*p<0.05*).

**Fig. 8:**
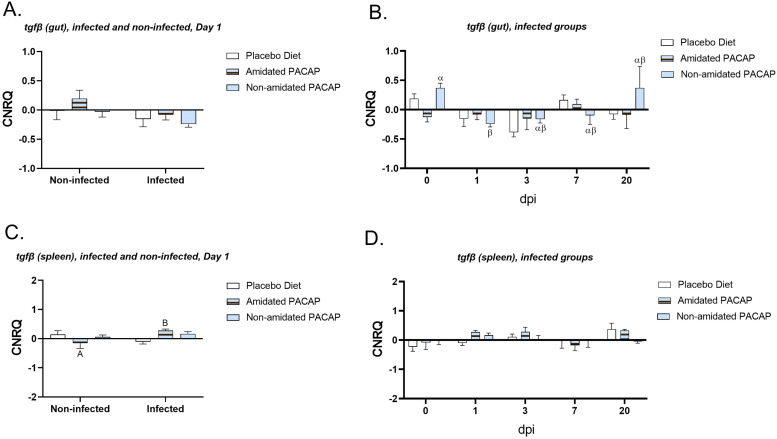
Calibrated normalized relative quantity of gut (A) and spleen (C) *tgfß* expressions of Atlantic salmon (*Salmo salar*) at Day 1 are shown with bars. Bars show the mean ± SEM. Calibrated normalized relative quantity of gut (B), spleen (D) *tgfß* expressions of infected Atlantic salmon (*Salmo salar*) at all time points are shown with bars. Letters denote significant group differences (*p<0.05*).

**Fig. 9:**
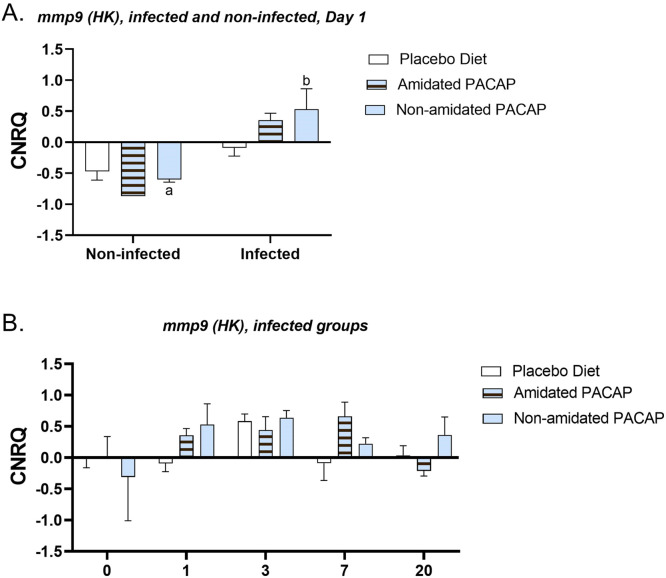
Calibrated normalized relative quantity of head kidney *mmp9* expressions of infected and non-infected Atlantic salmon (Salmo salar) on Day 1(A) and the *mmp9* expression of infected groups (B) are demonstrated with bars at all time points. Bars show the mean ± SEM. Letters are denoted as a significant difference between the two groups.

Comparing the expression of *hepcidin* between infected and non-infected groups in the spleen and head kidney on Day 1 (Fig. 7A and C) indicated a significant upregulation in the infected groups for both amidated (*p* *=* *0.0021*) and non-amidated PACAP (*p* *=* *0.0017*) in spleen. However, there was no difference between the two groups in the head kidney across the three diets at 1 dpi. When examining *hepcidin* expression in the infected groups at all time points for all treatment groups, it was found that within the first 24 h, there was a significant upregulation in *hepcidin* expression in spleen samples for both amidated (*p* *=* *0.0046*) and non-amidated PACAP (*p* *=* *0.035*) (Fig. 7B). A similar trend was observed for *hepcidin* expression in head kidney; however, a 2-way ANOVA analysis was not possible due to lack of detectable expression in some experimental groups (Fig. 7D). The expression of *tgfß* was compared between infected and non-infected groups in gut and spleen samples at the first 24 h (Fig. 8A and C). While there was no significant difference in the intestinal tissues, the spleen showed a significant upregulation of *tgfß* expression after infection with *Y. ruckeri* in the amidated PACAP group (*p* *=* *0.039*). Further assessment of *tgfß* expression in the guts of infected fish over 20 days post-infection revealed that at 1 day post-infection (dpi), non-amidated PACAP led to a significant downregulation (*p* = 0.018) (Fig. 8B). The evaluation of *mmp9* expressions in head kidney samples at 1 dpi revealed a significant upregulation in the infected non-amidated PACAP group compared to the non-infected group (Fig. 9A, *p = 0.006*). In contrast, no significant differences were observed in *mmp9* expression in the head kidney among the infected groups at any of the time points (Fig. 9B). There were no significant differences in the expression of *tnfα* and *il6* in the spleen between infected and non-infected groups on day 1 (see Sup. Figures 1 and 2A). Additionally, over time, no significant differences were observed in the infected groups across all diets (see Sup. Figures 1 and 2B). Furthermore, examining the head kidney tissues for *il6, c3a, and tapasin* expressions between infected and non-infected groups on day 1 across three diets (see Sup. Figs. 1C, 3A, and 4A) and assessing the infected groups over time for all diets (see Sup. Figs. 1D, 3B, and 4B) suggested no significant differences in any of these analyses.

### Histology examination and scoring

The results obtained from the observation and scoring of the histological lesions for both intestine and kidney samples demonstrated that regardless of the diets, there were no signs of inflammation, necrosis, bacteria, or other histological damage (i.e., villi edema, fusion, lymphangiectasia, and hyperplasia of goblet cells) at 0 and 1 dpi. However, there was some degree of (focal mild and multifocal moderate) inflammation in all treatment groups in the tunica muscularis layer of the intestines at 3, 7 and 20 dpi, which had increased in severity by 20 dpi. Furthermore, statistical analysis of the scoring of histological lesions in the tunica muscularis of the intestine of groups exposed to *Y. ruckeri* showed no significant differences among the treatment groups (Sup. Figure 6, 7). The kidney samples were observed for inflammation, necrosis, bacteria, and cell infiltration, but no signs of these clinical changes were recorded in any treatment group at any time point.

### IL1ß and IFN-γ quantification by sandwich ELISA

The serum protein concentration of IL-1β (pg/mL) was measured over the course of the study for both infected and non-infected salmon (Fig. 3D, E and F). At 1 dpi, a significant difference was observed between the amidated and non-amidated groups within the non-infected category (Fig. 3E, p = 0.017). Additionally, the serum protein concentration of IL-1β in infected fish showed significantly higher levels 3 dpi compared to 1 dpi for the placebo diet (Fig. 4D, *p* = 0.04). When quantifying IFN-γ protein in serum at 1 dpi, no significant differences were found between the infected and non-infected groups (Sup. Figure 1C). Likewise, the levels of IFN-γ protein in the serum of infected fish at 1 and 3 dpi across all diets did not show significant differences (Figs. 6C).

### Determination of antigen-specific IgT and IgM levels by indirect ELISA

**Fig. 10:**
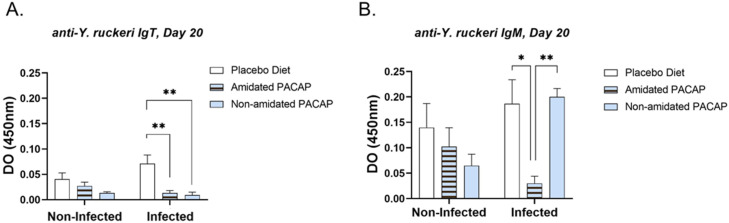
anti-*Y. ruckeri* IgT (A) and anti-*Y. ruckeri* IgM (B) were determined by indirect ELISA of non-infected and infected Atlantic salmon with *Y. ruckeri*. Bars show the mean ± SEM. Asterisks represent significant differences between the two groups (*P < 0.05, **P < 0.01, ***P < 0.001, ****P < 0.0001).

The detection of IgT protein in serum at 20 dpi for both infected and non-infected groups across all diets (Fig. 10A) revealed that infected fish receiving amidated PACAP showed significantly lower levels of IgT (*p* *=* *0.005*) compared to those on a placebo diet. Similarly, infected fish on a non-amidated PACAP diet also exhibited lower IgT levels (*p* *=* *0.002*) compared to the placebo group. Moreover, a comparison of IgM levels (Fig. 10B) in serum at 20 dpi of infected and non-infected fish revealed significant differences between the amidated group and both non-amidated infected fish (*p* *=* *0.0061)* and placebo (*p* *=* *0.012*) treated salmon.

## Discussion

In this study, an improved survival rate of fish fed the amidated form of PACAP compared to the groups that received commercial diets was observed following the challenge with *Y. ruckeri*, which may suggest the protective impacts of the PACAP in teleost fish after 28 days of feeding. After the challenge, there was a significant reduction in feed consumption across all groups for several days. However, the fish fed with amidated PACAP feed experienced significantly greater weight gain. Additionally, gaining a significant amount of weight in amidated PACAP treated groups suggested that this formulated diet was a palatable fish feed and had a meaningful impact on growth, regardless of infection. One of the concerns about therapeutic treatment with PACAP and administration through feed, which is more applicable in fish farm practice, is its short half-life period in the body. Several human studies have estimated the half-life of PACAP to be around 3–10 min [31,32]. However, this may be extended in teleosts due to their lower hepatic capacity and metabolism in detoxification. By C-terminal amidation of peptides, they become more protected against exopeptidases, which can extend the amidated peptides’ half-life [33], and it can enhance the antimicrobial efficacy of peptides, such as the peptide modeline-5, against *Escherichia coli* [34]. This fact agrees with the mortality results obtained. In previous work, PACAP-38, a potent immunosuppressant, had shown potent sterilizing activity against both Gram-negative and positive bacteria [35]. Unfortunately, our qPCR analysis of head kidney tissue was not able to detect *Y. ruckeri* and quantify the level of infection reliably in infected fish, making assessment of bacterial load impossible. Although the cDNA pools of the samples showed a higher bacterial load, each individual infected sample indicated a lower abundance of *Y. ruckeri*, and the primer design and choice of DNA strand are essential for precisely measuring the bacterial loads by real-time PCR [36]. The confirmation of *Y. ruckeri* from multiple tissues through MALDI-TOF of moribund along with external signs of infection, indicates that the bacterial infection caused stress and ultimately led to mortality. However, no significant histological lesions were detected in either the kidney or intestine samples.

It is generally agreed that the gill, specifically pavement cells of the gill lamellae, is the initial site of infection by *Y. ruckeri* [[37], [38], [39]]. The bacteria adhere to mucus on the gill lamellae and invade the cell through surface receptors, thereby initiating a signaling cascade which results in phagocytosis of the bacterium into the cell where it can survive and replicate [40]. The bacterium then enters the circulation and spreads to other internal organs [39]. *Y. ruckeri* can also reside latently in the intestine [38]. PACAP has been shown to have no direct effect on the adhesion of enteric bacterial pathogens to intestinal cells. However, it can counteract endotoxin (lipopolysaccharide (LPS))-induced increased expression of *il8* and *cxcl-1* in mammals [41]. Bacterial endotoxins, or LPS, found in Gram-negative bacteria, play a vital role in the initiation of responses that can lead to endotoxic shock. The immunomodulatory, anti-shock effect of PACAP on experimental endotoxin has been reported in different animal models of acute inflammatory conditions [42]. An investigation of the PAC1 receptor's role in neutrophil recruitment and acute phase responses during septic shock in mice confirmed that the PAC1 receptor plays an anti-inflammatory role by reducing the production of *il6* [43]. The vasoactive intestinal polypeptide (VIP) and PACAP share 68 % sequence similarity, resulting in PACAP-38 being able to bind to the same receptors as VIP, resulting in both being candidates for multitarget therapy of endotoxic shock [44]. PACAP has also been shown to increase the survival rate of mice with septic peritonitis by inhibiting inflammation via NF-κB activation [45].

Typically, bacterial infections in fish lead to an increased expression of *ilß* and *il10* in the first few days after exposure*; ilß* and *il10* are known to be pro-inflammatory and anti-inflammatory cytokines, respectively [46,47]. In this feeding trial, the expression of *ilß* was upregulated in infected fish across all three diets between 1 and 3 dpi in the gut. However, this increase was more significant in the placebo groups. This outcome suggests that both amidated and non-amidated PACAP-formulated feeds helped control inflammation to some extent and played a role in reducing mortality in the treatment groups. Moreover, Fajei et al. indicated that PACAP-38 may possess anti-inflammatory properties with the same immunological mechanisms [21]. Prior research on rats indicated that PACAP-38 inhibits acute neurogenic and non-neurogenic inflammatory processes [48]. In this project, similar to Frenette et al. [26], IL-1ß serum concentrations were elevated in the infected fish, particularly within the first 24 h post-infection. However, this increase was not observed in the groups that consumed PACAP-formulated feed. The expression of *il10* in the gut, spleen, and head kidney showed a similar trend from 0 to 3 dpi. There was a decrease at 1 dpi, particularly marked by a significant downregulation in the non-amidated PACAP group within the gut. By 3 dpi, there was an upregulation in all three tissues, with a significant increase observed specifically in the spleen. In fish tissues, *il10 expression* is widely distributed and increases in response to bacterial and viral infections [49,50]. It is a pleiotropic cytokine with immunoregulatory roles, which are intricate to evaluate in all species of teleost fish [50,51]. Similarly, the anti-inflammatory activity of PACAP was also observed in human chronic pancreatitis, where an enhanced level of *il10* production was observed in activated macrophages [52]. An increase in the total number of caliciform cells within the intestinal villi was also observed in rainbow trout fed a dietary supplement of PACAP, suggesting a possible impact on digestibility and defense against invading pathogens [53], such as *Y. ruckeri*. These findings imply that although PACAP does not directly affect bacterial adhesion, it may impact intestinal cells' cytokine production after exposure to endotoxins. This may explain some of PACAP's anti-inflammatory properties in the digestive tract [41].

Some studies have shown that synthetic PACAP-38 possesses antibacterial activity against various bacteria. Specifically, PACAP-38 minimized the growth of *Burkholderia cenocepacia* (J2315) with a reported MIC of 10 μg/mL [54]. Furthermore, Rainbow trout *(Oncorhynchus mykiss)* infected with *Y. ruckeri* exhibited an upregulation of the transcripts PACAP-38, PRP/PACAP, and VPAC2 receptors in the spleen compared to healthy groups [53]. Also, synthetic PACAP-38, at a concentration of 50–100 µM in TSB, directly impacted the inhibition of *Y. ruckeri* growth by 60 % [44]. Both Velazquez et al. 2023 [53] and Semple et al. 2019 [44] also demonstrated a significant reduction in bacterial growth of *Y. ruckeri* and *F. psychrophilium*, respectively, in the monocyte/macrophage-like Rainbow trout cell line RTS11 pre-treated with variants of PACAP.

PACAP has also been shown to have an immunomodulatory effect, as demonstrated in the Rainbow trout RTgutGC cell line infected with *Aeromonas salmonicida* where *il1ß* and *il8* expression was significantly downregulated, and *tgfß* expression was upregulated after PACAP treatment [54]. Rivera-Méndez et al. 2024 [28] proposed that PACAP may prevent inflammation in the gut and improve general fish health, and indeed, *tgfß* has been shown to suppress the T-cell response in teleost fish [55]. In this research, *tgfß* expression within 1 dpi was significantly upregulated in infected fish treated with amidated PACAP in the spleen. In the gut non-amidated PACAP group also exhibited a significant change in *tgfß* expression between 0 and 20 dpi. Some earlier studies investigating effective and applicable treatments and vaccinations for ERM have demonstrated different pathways activated by vaccination and pathogenic challenges [56,57]. In this study, immunological induction of *hepC* was observed in the spleen at 1 and 3 dpi. Similarly, Deshmukh et al. 2013 also reported a significant increase in *il10* and *il1ß* in the head kidney in non-immunized groups [56]. Although the upregulation of *il1ß* expression was not statistically significant among the study groups, its elevation was noted, especially in the first-week post-infection. Expression of *il10, il1ß,* and *hepC* in the spleen and gills of Rainbow trout in response to ERM vaccination was significantly elevated in the 1–7 days post-vaccination (dpv) with *il1ß,* and *hepC* downregulated by 14 dpv [57]. This trend agrees with the data presented in this study. Different cells have the capability of releasing *il1ß* when pathogen-associated molecular patterns (PAMPs) such as LPS activate host pattern recognition receptors (PRPs), thereby regulating the inflammatory responses in fish [58]. The upregulation in the expression of *il1ß* was significant, and the data suggest that the significantly lower mortality can result from the inflammatory regulation by *il1ß* in the PACAP-treated groups*.* Also*, il10,* impacts on the immune system, results in the differentiation of B cells, boosting antibody production [59], plus the elevation of its expression in PACAP-treated groups can affect a rapid immune response against infection. Hepcidin, a small cysteine-rich protein with antimicrobial features, has a crucial role in iron homeostasis, and it can be isolated relatively simply from the gill of fish challenged with bacteria [57]. Gene expression analysis of spleen and head kidney in African catfish (*Clarias gariepinus*) post-treatment with PACAP-38 and exposed to *Pseudomonas aeruginosa* showed a significant up-regulation in *il1ß* expression within 2 dpi as well [60]. In Atlantic salmon *(Salmo salar)* liver tissue, *hepC* expression significantly increased 24 h post-bacterial challenge with a genetically attenuated strain of *A. salmonicida*. On the other hand, in fish starved for 28 days prior to the bacterial challenge, the increase in *hepC* gene expression was more profound [61]. Also*, hepC* has demonstrated a significant increase in the liver of Nile tilapia *(Oreochromis niloticus*) infected with *Streptococcus iniae* [62].

The expression of *mmp9* in the head kidney of infected fish that received non-amidated PACAP showed a significant increase at 1 dpi compared to uninfected groups. Although the elevation of *mmp9* levels in PACAP-treated groups during the study was not significant at all time points, it aligns with its role in cell repair and regeneration as well as its involvement in inflammatory responses to bacterial infections in fish [63]. The lack of tissue damage also supports this, and the observation of organized tissue patterns, which agrees with other work that has demonstrated treatment with PACAP-38 could alleviate the pathological symptoms of ileitis in a mouse model of inflammatory bowel disease and cell migration [64]. The *mmp9*, and mmp-inhibitors like (TIMP-1, TIMP-2) are often elevated in the plasma of patients with sepsis. However, studies examining the association between an increase in *mmps* and the severity of disease have shown that MMP9 is negatively correlated with disease severity. Further, MMP9 deficiency in mice results in abdominal sepsis and reduces leukocyte recruitment [[65], [66], [67]]. The regulatory mechanisms of MMP9 and other *mmps* are challenging to elucidate in infectious diseases since many factors can influence them, but their study in relation to PACAP-induced protection is further warranted.

The IgT level was significantly decreased in the serum of infected fish treated with PACAP-formulated feed at 20 dpi. Like mammalian IgA, IgT is a teleost-specific antibody isotype that is essential for fish mucosal immunity [68]. IgM is more prevalent in serum, whereas IgT is mostly expressed in mucosal tissues such as the intestine and gills [69]. Serum IgT levels in Atlantic salmon are 100–1000 times lower than serum IgM levels [70]. The immunoglobulins are typically elevated in response to infections. A significant decrease in immunoglobulin levels, particularly IgT, in the PACAP-treated groups respect placebo diet groups after 20 days, indicates the resolution of infection. This resolution contributed to a reduction in mortality within these PACAP-treated groups.

In summary, the results of this study showed that PACAP-supplemented diets, especially with amidated PACAP, reduced mortality and initiated the immunological pathways to resolve the infection, as indicated in both immunological and histological analyses. Future work should assess different dosages and feeding regimes of amidated PACAP to determine optimal dosing and strategic therapy to maximize impacts on infection and survival.

## CRediT authorship contribution statement

**E Fajei:** Writing – review & editing, Writing – original draft, Visualization, Software, Project administration, Methodology, Formal analysis, Conceptualization. **L Rivera Méndez:** Writing – review & editing, Visualization, Formal analysis. **SK Whyte:** Writing – review & editing, Supervision, Project administration, Methodology, Formal analysis. **J Velazquez:** Writing – review & editing, Project administration. **P Dantagnan:** Writing – review & editing. **M Soto-Davila:** Writing – review & editing. **T Rodríguez-Ramos:** Writing – review & editing, Methodology, Formal analysis. **E Proskos:** Visualization, Formal analysis. **B Dixon:** Writing – review & editing, Validation, Supervision, Methodology. **Y Carpio:** Writing – review & editing, Visualization, Formal analysis. **M Estrada:** Writing – review & editing. **MD Fast:** Writing – review & editing, Supervision, Methodology, Formal analysis.

## Declaration of competing interest

The author is an Editorial Board Member/Editor-in-Chief/Associate Editor/Guest Editor for this journal and was not involved in the editorial review or the decision to publish this article.

## Data Availability

Data will be made available on request.

## Appendix Supplementary materials

Image, application 1
