# Supplementary figures and images for: Investigation of two different PACAP-38 (Pituitary Adenylate Cyclase Activating Polypeptide) formulated feeds on Atlantic salmon (Salmo salar) immune responses with Enteric Red Mouth disease (Yersinia ruckeri)
Source: Comp Immunol Rep. 2025 Mar 30;8:200221. doi: 10.1016/j.cirep.2025.200221 (PMC12000741; doi:10.1016/j.cirep.2025.200221)

## Slide 1
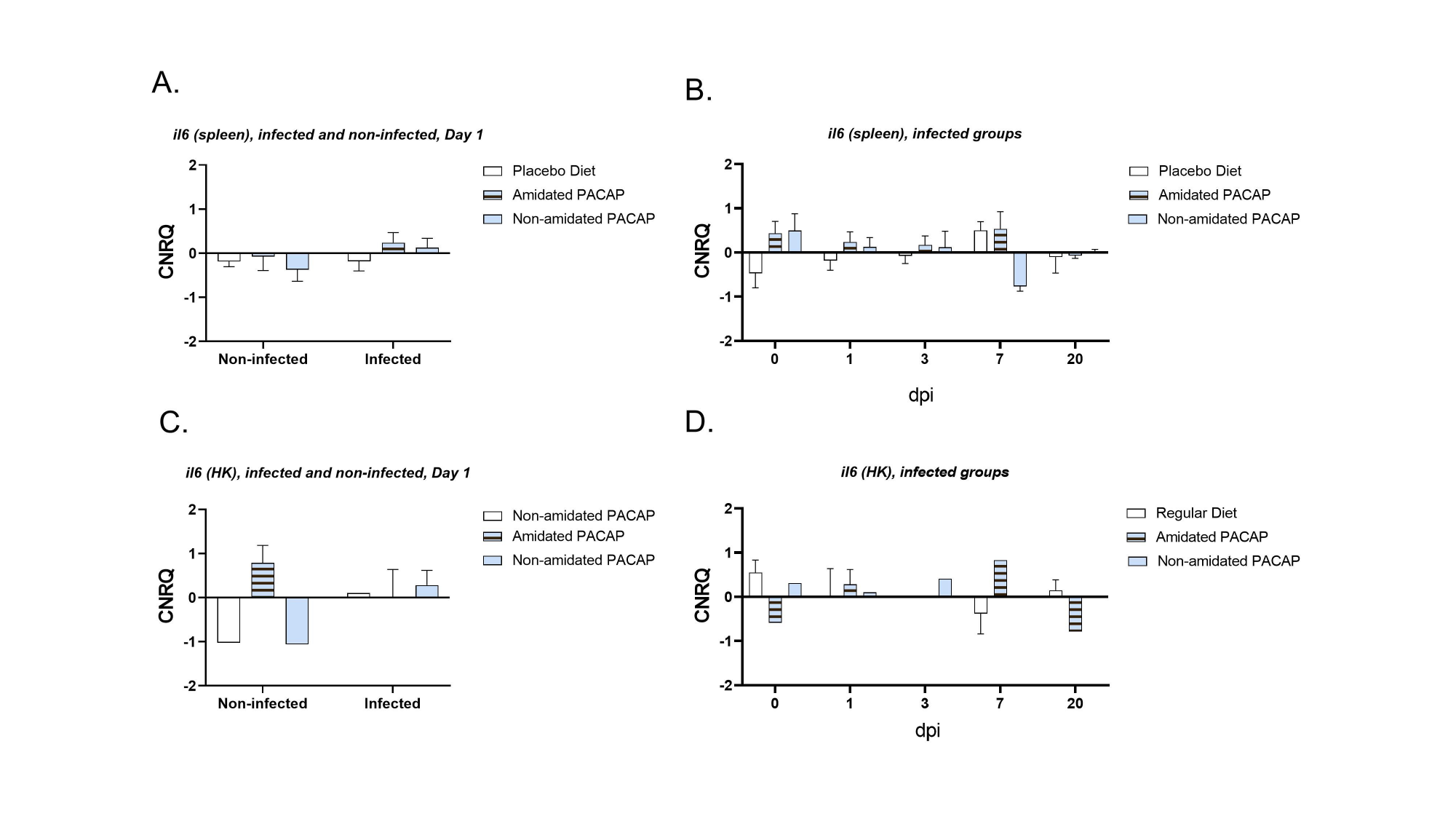

## Slide 2
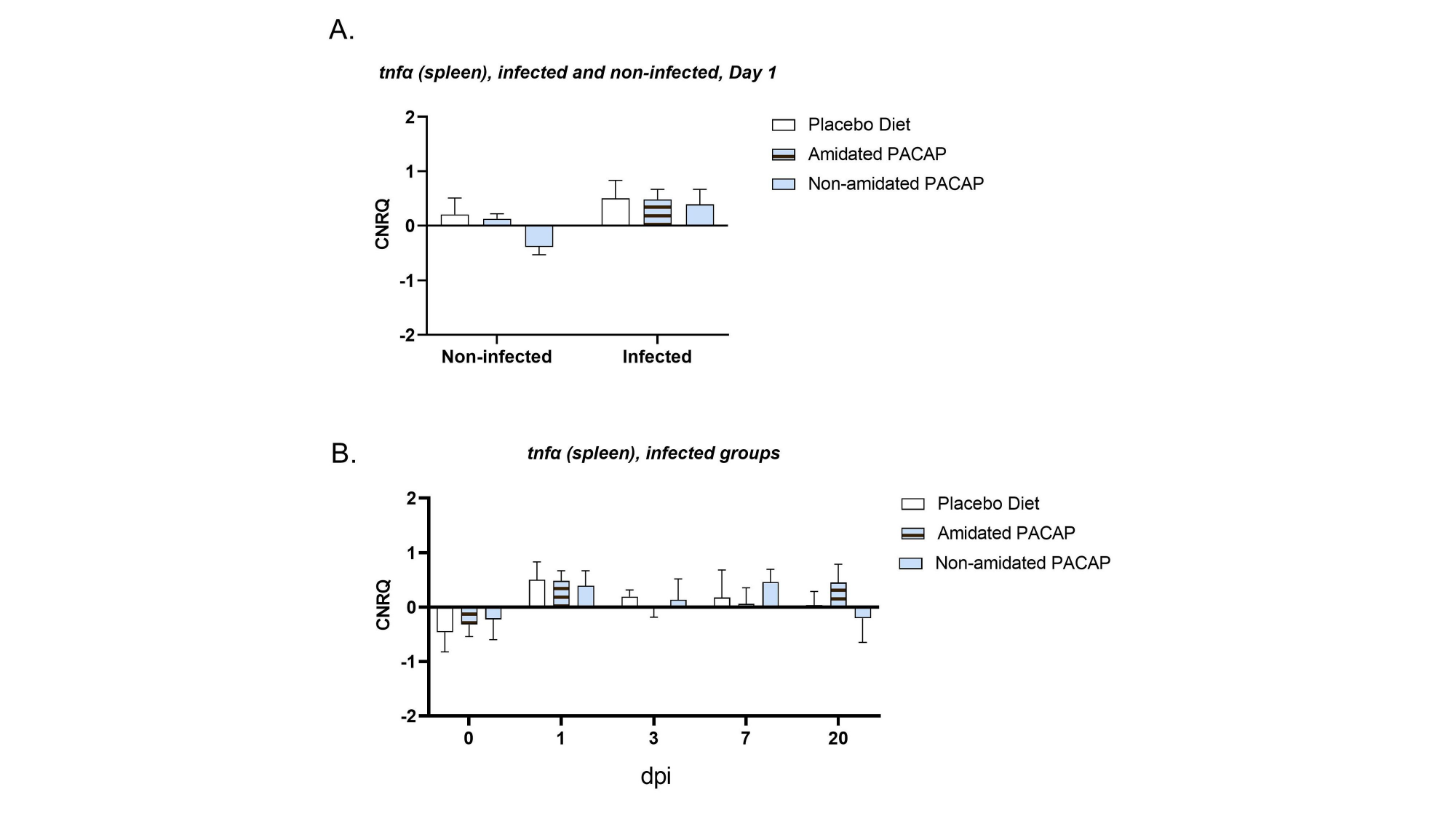

## Slide 3
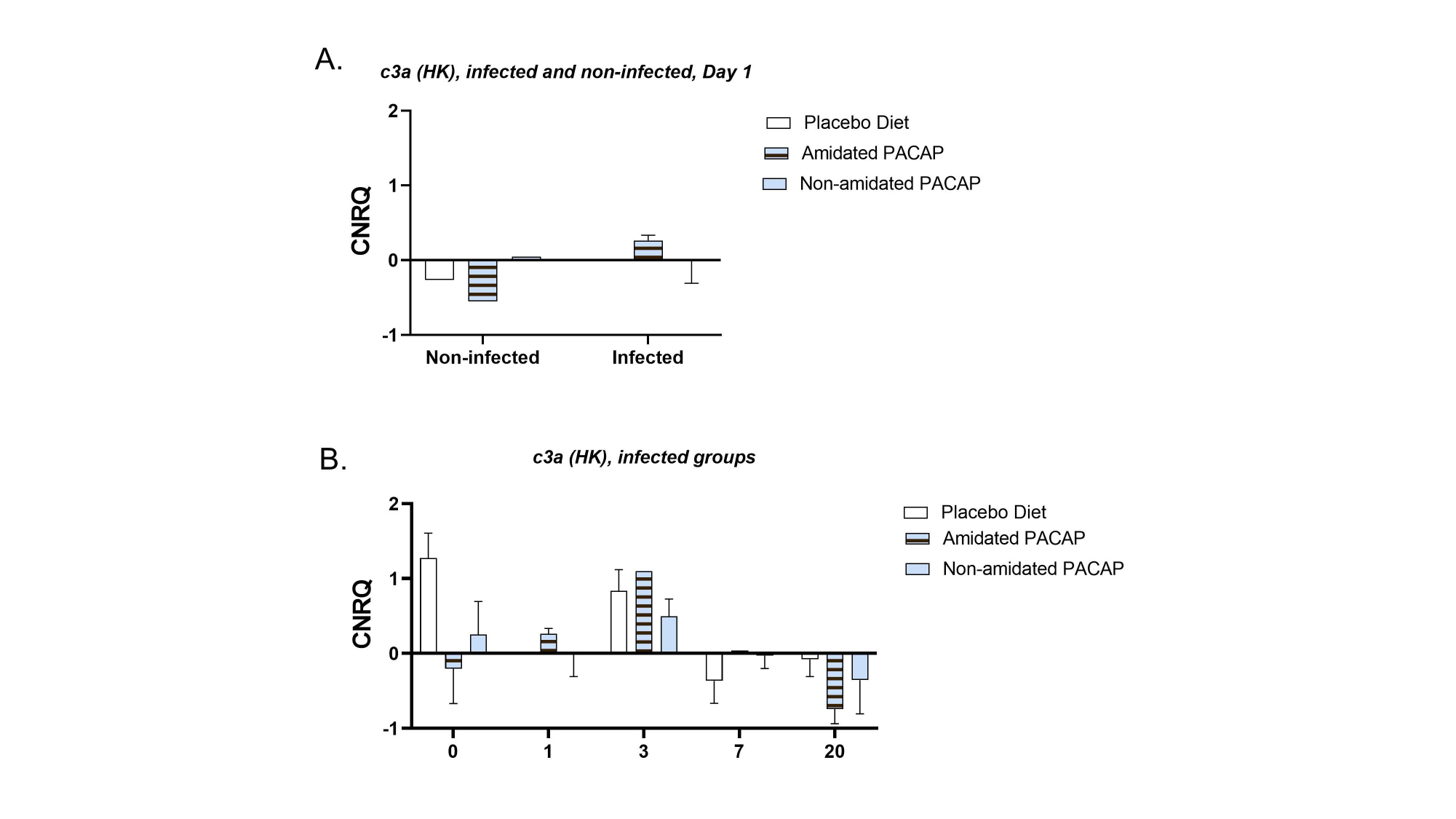

## Slide 4
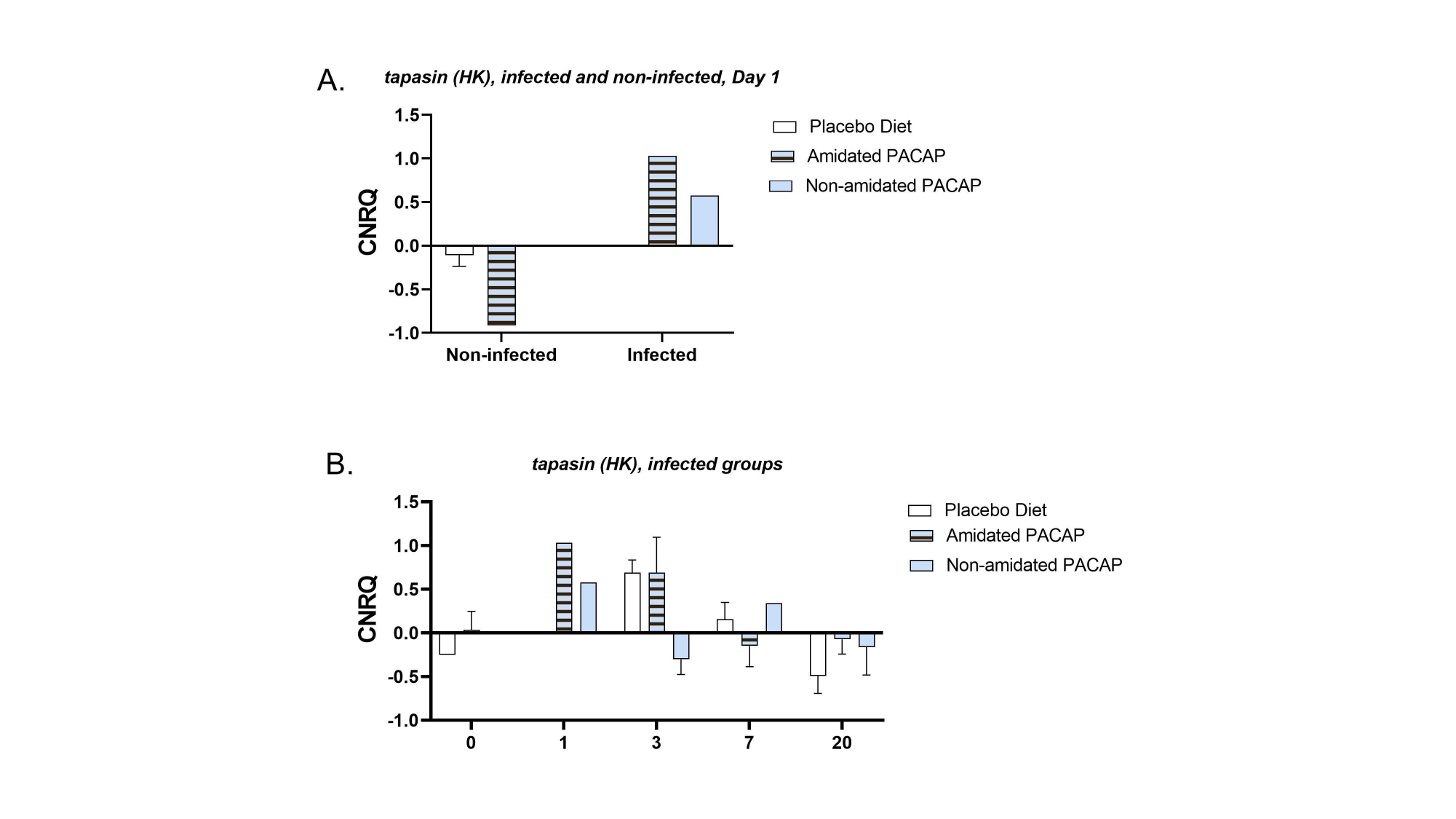

## Slide 5
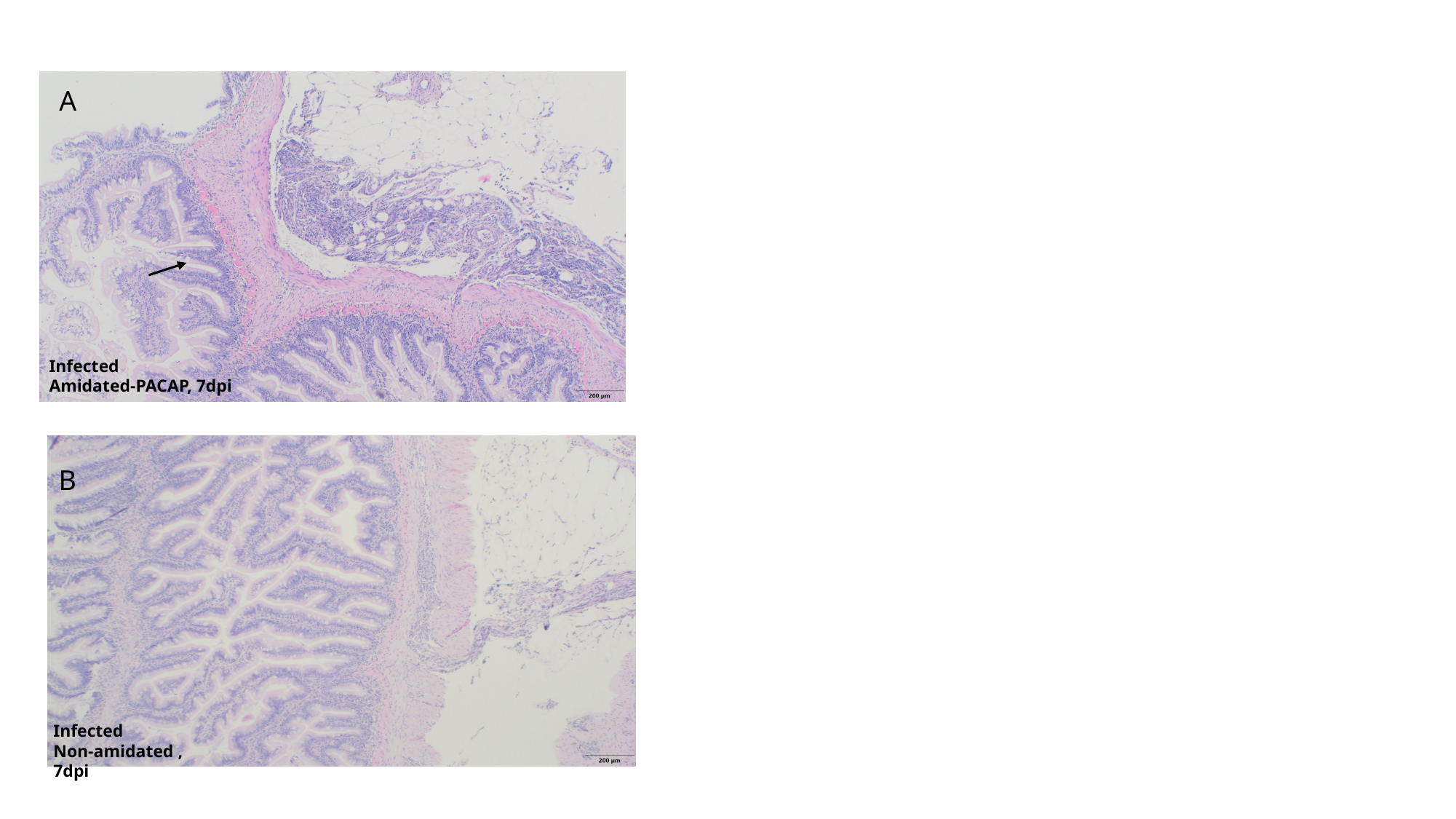

A
Infected
Amidated-PACAP, 7dpi
B
Infected
Non-amidated , 7dpi

## Slide 6
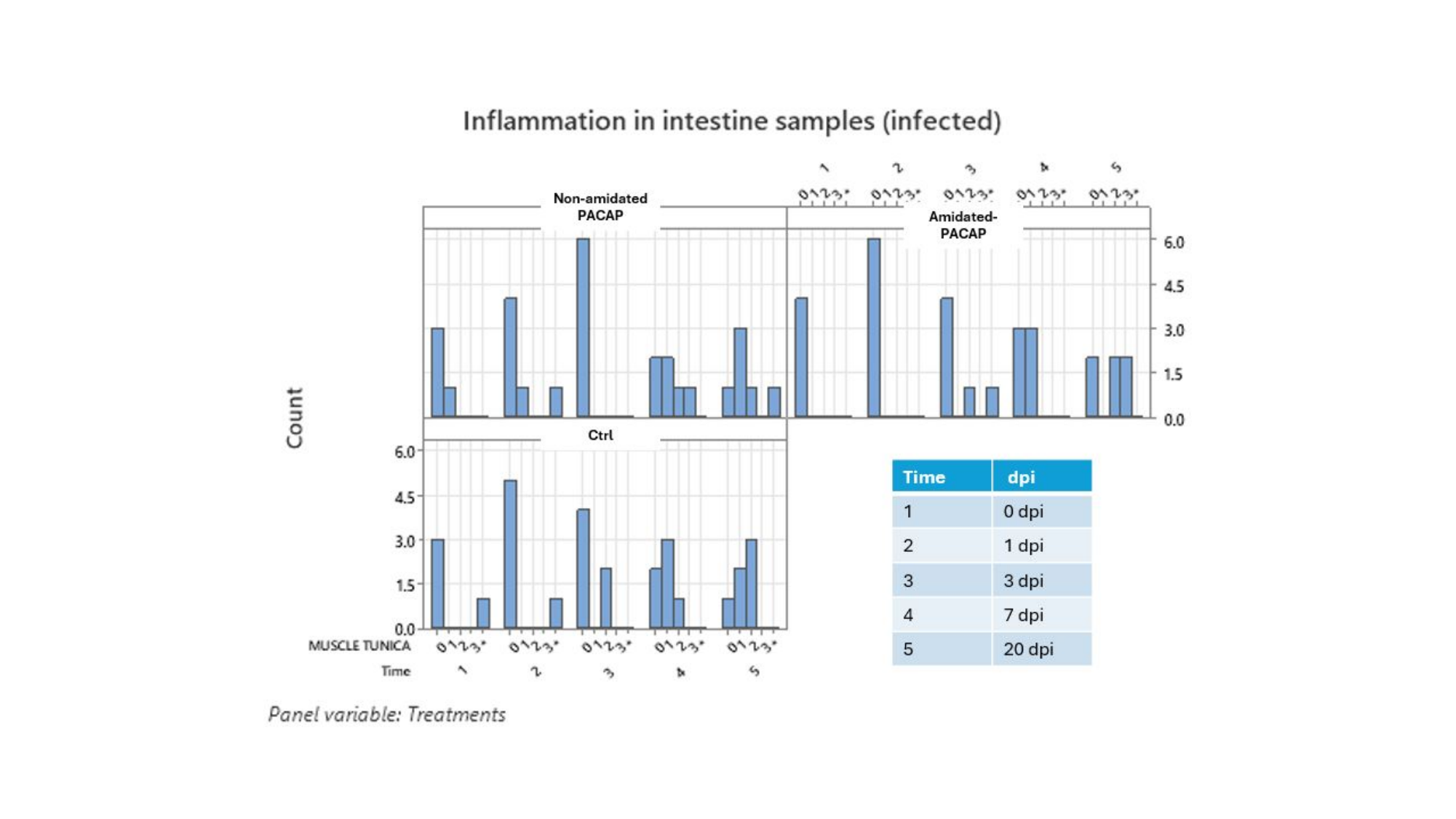

Supplement: Supplementary file 1 [file mmc1.pptx]
